# Initiating an epilepsy surgery program with limited resources in Indonesia

**DOI:** 10.1038/s41598-021-84404-5

**Published:** 2021-03-03

**Authors:** Muhamad Thohar Arifin, Ryosuke Hanaya, Yuriz Bakhtiar, Aris Catur Bintoro, Koji Iida, Kaoru Kurisu, Kazunori Arita, Jacob Bunyamin, Rofat Askoro, Surya Pratama Brilliantika, Novita Ikbar Khairunnisa, Zainal Muttaqin

**Affiliations:** 1 Department of Neurosurgery, Faculty of Medicine, Diponegoro University, Semarang City, Central Java Province Indonesia; 2 Department of Neurosurgery, Graduate School of Medical and Dental Sciences, Kagoshima University, Kagoshima City, Kagoshima Prefecture Japan; 3 Department of Neurology, Faculty of Medicine, Diponegoro University, Semarang City, Central Java Province Indonesia; 4 Department of Neurosurgery, Graduate School of Biomedical and Health Sciences, Hiroshima University, Higashihiroshima City, Hiroshima Prefecture Japan

**Keywords:** Health services, Neurology

## Abstract

To share the experiences of organizing the epilepsy surgery program in Indonesia. This study was divided into two periods based on the presurgical evaluation method: the first period (1999–2004), when interictal electroencephalogram (EEG) and magnetic resonance imaging (MRI) were used mainly for confirmation, and the second period (2005–2017), when long-term non-invasive and invasive video-EEG was involved in the evaluation. Long-term outcomes were recorded up to December 2019 based on the Engel scale. All 65 surgical recruits in the first period possessed temporal lobe epilepsy (TLE), while 524 patients were treated in the second period. In the first period, 76.8%, 16.1%, and 7.1% of patients with TLE achieved Classes I, II, and III, respectively, and in the second period, 89.4%, 5.5%, and 4.9% achieved Classes I, II, and III, respectively, alongside Class IV, at 0.3%. The overall median survival times for patients with focal impaired awareness seizures (FIAS), focal to bilateral tonic–clonic seizures and generalized tonic–clonic seizures were 9, 11 and 11 years (95% CI: 8.170–9.830, 10.170–11.830, and 7.265–14.735), respectively, with *p* = 0.04. The utilization of stringent and selective criteria to reserve surgeries is important for a successful epilepsy program with limited resources.

## Introduction

Epilepsy is the most common neurological disorder affecting people of all ages and classes^1^. Interestingly, the prevalence of epilepsy differs between developed and developing countries, in which the latter has a higher prevalence in both urban and rural settings^2^. Approximately 50 million people worldwide suffer from this disorder, and 80% among them reside in developing countries with limited available resources^3^. The remainder of these 40 million people are unable to obtain appropriate treatment and, as a result, experience significant morbidity owing to seizures^4^. Furthermore, they still face psychosocial implications from stigma and prejudice due to stereotypes and negative attitudes towards epilepsy, especially in developing countries^5^.

Anti-seizure drug (ASD) intervention is the mainstay for care for people with epilepsy; however, more than 30% of people with epilepsy experience frequent hallucinations through the use of sufficient ASDs^6^. Surgery is one of the essential treatment choices for patients whose ASD medication fails to stop seizures and/or to achieve lasting relief from seizures. The effectiveness of epilepsy surgery relies on the successful selection of appropriate surgical candidates based on available information and technologies. Nevertheless, resources are limited for the vast majority of patients with drug-resistant epilepsy (DRE), who live mostly in developing countries.

Epilepsy surgery in Indonesia started in 1999 under the direction of mentoring universities from Japan. We have now implemented intracranial electrode monitoring to the sum of 62 surgeries up until 2017. We share our experience in establishing an epilepsy surgery program in Indonesia, its challenges, and how we overcame those issues.

## Methods

### Surgical indication and presurgical evaluation

We report our experience with patients who underwent epilepsy surgery in our neurosurgical centres in Semarang, Indonesia (Kariadi Hospital and the affiliated Diponegoro Hospital) between July 1999 and December 2017. We divided this timespan into two periods based on resource availability: the first period (1999–2004) and the second period (2005–2017).

In the first period, the surgical indication for patients with drug-resistant temporal lobe epilepsy (TLE) was considered primarily based on whether they had a seizure-related lesion. In addition to reviewing past treatment and a detailed history of medical and neurological examinations, neuropsychological tests, and psychiatric evaluations, all patients received scalp electroencephalogram (EEG) while awake and asleep and magnetic resonance imaging (MRI) scans of at least 0.5 T or more. The MRI series included regular orientation of 5-mm slices at T1WI and T2WI and fluid-attenuated inversion recovery (FLAIR). Invasive recordings and operations on patients without MRI confirmed lesions were not feasible during this time.

In the second period (2005–2017), the long-term monitoring of EEG recordings (Video EEG) was adopted for the presurgical evaluation, and intervention was aimed at different types of seizures. The phase one study was basically the same as in the first period. As an advanced analysis, long-term EEG and PET investigations were performed in difficult cases using conventional methods^7^. The Wada test was also performed on some patients. Phase two included invasive video EEG monitoring with subdural electrodes. Functional mapping was performed using subdural electrodes.

A reassessment was scheduled or alternative treatments were recommended for patients in cases where preoperative investigations were not consistent. During phase three (the intraoperation phase), we analysed the electrocorticogram (ECoG) of interictal activity. Awake functional mapping and/or the somatosensory evoked potential test was performed if necessary.

### Surgical procedures

In the first period, most patients underwent anterior temporal lobectomy (ATL). In the second period, we introduced selective amygdalohippocampectomy (SAH) for patients with unilateral dominant mesial temporal lobe surgery (TLE). At this point, we started to treat extratemporal lobe epilepsy, and we performed a variety of surgical procedures. Cortical resection and multiple subpial transection (MST) were used for extratemporal neocortical epilepsy in non-eloquent and eloquent foci, respectively. Anterior callosotomy was indicated for infantile hemiplegia, Lennox–Gastaut syndrome, multifocal bilateral epilepsy and drop attacks for palliative surgical procedures.

### Postsurgical evaluation

A pathological analysis of haematoxylin–eosin (H&E)-stained resected samples was started in the second period. Several additional studies were applied to the hippocampus. Upon postoperative treatment, AEDs were gradually reduced after a duration from six months to one year, in agreement with the patient who underwent follow-up EEG every six months to one year. Postoperative seizure results were measured using the Engel Outcome Scale (EOS) for patients who underwent surgery at least 2 years after the procedure.

### Study design, participants, and ethical declaration

We recorded the long-term outcomes and patterns of seizure remission and relapse in 589 consecutive participants who underwent epilepsy surgery between July 1999 and December 2017. Based on the availability of resources, this timespan was divided into two periods: the first period (1999–2004) and the second period (2005–2017). Two Indonesian consultant neurosurgeons specializing in epilepsy (Z.M. and M.T.A.) conducted the operations with the direct supervision by Japanese mentors (K.A., R.H., K.K., and K.I.) for the first 15 cases in the first period. Clinical history, examination findings, seizure semiology, interictal and ictal EEG, MRI results, neuropsychological and psychiatric assessments, and clarity of the epileptogenic zone location were examined as potential predictors of neurological deficits. We also performed additional presurgical evaluations, i.e., intracarotid amytal, FDG-PET, ECoG, and subdural grid EEG. The optimum surgical approach was decided to provide the best chance of freedom from seizures with the lowest risk of complications using the general principles of presurgical assessments previously mentioned. Furthermore, information on the yearly seizure status was obtained from a review of contemporaneous notes from Kariadi Hospital and from the records of other hospitals attended by the patient. Thereafter, postoperative seizure results were measured using the Engel Outcome Scale (EOS) for individuals who had undergone an operation 2 years prior to the assessment.

All procedures performed in studies involving human participants were in accordance with the ethical standards of the Medical Faculty, Diponegoro University and Kariadi Hospital Ethical Research Committee and with the 1964 Helsinki Declaration standards. This study was approved by the Joint Ethics Committee of the Kariadi General Hospital (number 122/EC/KEPK-RSDK/2019). Written informed consent was obtained from all patients prior. For patients under the age of 18 years, informed consent was obtained from a parent and/or legal guardian.

### Statistical analysis

Statistical analysis was performed using Statistical Package for the Social Sciences, version 22 (SPSS v22.0, IBM, USA). Differences between variables were tested with the non-parametric chi-square test. Survival analysis with Cox proportional hazards regression was used to evaluate the total time-to-event outcomes and compare the postoperative seizure-free time (Engel 1a and 1b). Hazard ratios (HRs) (and 95% CIs) were calculated for the operation type, aura, epileptogenic source and type of surgery during the first and second periods, as well as for the overall group. The Kaplan–Meier method was used to estimate the proportion of individuals who remained seizure-free at various timepoints (years), while the median survival time was reported as the 95% CI, with a 3-, 5- and 10-year chance of being seizure-free during follow-up.

## Results

Five hundred eighty-nine consecutive patients were treated between July 1999 and December 2017. Among the 65 patients treated in the first period (Table 1), the most common seizure type was focal with impaired awareness and focal to bilateral tonic–clonic, with six cases of generalized seizures. The majority of the aetiologies were associated with hippocampal sclerosis, with three tumours diagnosed through MRI findings: two dysembryoplastic neuroepithelial tumours (DNTs) and one of unknown aetiology. Two non-lesional cases of TLE were recognized, demonstrating typical signs, although the interictal EEG was unable to identify lateralization. Therefore, subdural electrodes were inserted under mentor supervision, and the EEG was recorded for a few hours. The resulting electrocorticography (ECoG) demonstrated the lateralization of interictal spikes in one person and ictal spikes in the other. Most cases in the first period had an epileptogenic zone within the temporal lobes (Table 1).

**Table 1:** Characteristics of patients who underwent epilepsy surgery.

Characteristic	1st period		2nd period		Total
	N	%	n	%	
Number of patients	65	11.04	524	88.96	589
Sex					
Male	40	11.49	308	88.51	348
Female	24	9.96	216	89.63	241
Age (years)					
At seizure onset (average)	10.7		11.6		11.5
At surgery (average)	24.4		22.9		23.1
Seizure type					
Focal seizure	58	11.93	428	88.07	486
Focal impaired consciousness	33	11.79	247	88.21	280
Focal to generalized tonic–clonic	25	12.38	177	87.62	202
Others	0	0.00	4	100.00	4
eneralized seizure	6	18.18	27	81.82	33
Magnetic resonance imaging findings					
Normal	2	2.22	88	97.78	90
Single abnormality	62	14.00	381	86.00	443
Multiple abnormalities	1	25.00	3	75.00	4
Intracranial recordings	0	0.00	62	100.00	62
FDG-PET study	1	2.44	40	97.56	41
Epileptogenic zone					
Temporal lobe	56	13.73	352	86.27	408
Extratemporal lobe	2	5.71	33	94.29	35
Generalized	6	18.18	27	81.82	33

*FDG-PET* Fluorodeoxyglucose-positron emission tomography.

In the second period (Table 1), 524 patients were treated: 428 with focal seizures, among whom 247 and 4 had impaired awareness and aware seizures, respectively. In addition, 177 patients had focal-to-bilateral tonic–clonic seizures, and 27 had generalized seizures, while the presurgical analysis identified 86.27% of the epileptogenic focus in the temporal lobe.

This series recognized 90 patients with normal MRI findings during first and second periods. There were also 443 and 4 patients with single and multiple abnormalities, respectively (Table 1). Scalp EEG, MRI and seizure semiology were the primary presurgical evaluation tools used during the first period, while advanced assessments including long-term video EEG and PET were used during the second period (Table 2). The decision to proceed with surgery was reserved in 74 patients with normal MRI TLE. Subdural grid was placed in 11 patients monitored with long-term video EEG and 46 patients decided on LTSV. Furthermore, surgical outcome based on the multimodality evaluation was presented in Table 3. The underlying pathology was mostly mesial temporal sclerosis (MTS, 56.16%), as seen in Table 4.

**Table 2:** Modality used for the presurgical evaluation in the second period.

	N	Electroencephalography				MRI	FDG-PET	Wada test
		IIS	LTSV	IC	NL			
Temporal lobe epilepsy								
Abnormal MRI	279	57	159	19	43	279	9	0
Normal MRI	74	17	46	11	0	74	16	0
Extratemporal lobe epilepsy	33	33	0	13	0	33	2	1
Generalized epilepsy	20	17	0	2	0	20	0	0

*IIS* Interictal scalp, *LTSV* Long-term scalp video, *IC* Intracranial, *NL* No lateralization, *MRI* Magnetic resonance imaging, *FDG-PET* fFluorodeoxyglucose-positron emission tomography.

**Table 3:** Surgical outcome based on the multimodality evaluation.

Characteristic	Engel outcome scale							
	1st period (n)				2nd period (n)			
	I	II	III	IV	I	II	III	IV
Temporal lobe epilepsy with normal MRI	0	0	0	0	65	5	2	0
Scalp EEG + MRI	33	7	2	0	13	1	3	0
Scalp EEG + MRI + Video EEG					43	2	2	0
Scalp EEG + MRI + Video EEG + PET					14	1	0	0
Extratemporal lobe epilepsy	2	1	1	0	24	2	4	0
Scalp EEG + MRI	0	1	1	0	21	1	4	0
Scalp EEG + MRI + Video EEG					0	0	0	0
Scalp EEG + MRI + Video EEG + PET					0	0	0	0

*I* Class 1, *II* Class 2, *III* Class 3, *IV* Class 4, *EEG* Electroencephalogram, *MRI* Magnetic resonance imaging, *SPECT* Single-photon emission computed tomography, *PET* Positron emission tomography.

**Table 4:** Surgical outcome based on pathology.

MRI finding	Engel class					Total	%
	Ia	Ib	II	III	IV		
Abnormal gyrus	4	0	2	0	0	6	1.21
Arachnoid cyst	2	0	1	0	0	3	0.61
Astrocytoma	1	1	1	0	0	3	0.61
Arteriovenous malformation	2	0	0	0	0	2	0.40
Cavernoma	12	0	1	0	0	13	2.63
Cerebral dysgenesis	0	1	0	0	0	1	0.20
Cerebral contusion	0	1	0	0	0	1	0.20
Cortical dysplasia	27	0	2	3	0	32	6.46
Cystic lesion	2	0	0	0	0	2	0.40
Dysembryoplastic neuroepithelial tumour	6	0	0	0	0	6	1.21
Encephalomalacia	6	0	0	5	0	11	2.22
Epidermoid	1	0	0	0	0	1	0.20
Ganglioglioma	4	1	0	0	0	5	1.01
Gliosis	1	0	0	0	0	1	0.20
Hamartoma	0	0	0	1	0	1	0.20
Hemispheric Atrophy	10	0	3	2	0	15	3.03
Hippocampal Malrotation	1	0	0	0	0	1	0.20
Ischaemic/Infarct	2	0	0	0	0	2	0.40
Lesion	0	0	0	1	0	1	0.20
Mesial temporal sclerosis/hippocampal sclerosis	230	15	19	13	1	278	56.16
Porencephalic Cyst	10	2	0	0	0	12	2.42
Rasmussen	1	0	0	0	0	1	0.20
Sturge–Weber syndrome	1	0	0	0	0	1	0.20
Tuberous sclerosis complex	5	0	1	0	0	6	1.21
Ulegyria	1	0	0	0	0	1	0.20
Vascular lesion	0	0	0	1	0	1	0.20
Hemispheric Atrophy + Porencephalic Cyst	0	0	0	0	0	0	0.00
MTS/HS + Cortical Dysplasia	0	0	1	0	0	1	0.20
MTS/HS + Encephalomalacia	1	0	0	0	0	1	0.20
TSC + Ischaemia	0	0	0	0	0	0	0.00
Negative	73	2	6	5	0	86	17.37
Total	403	23	37	31	1	495	

*MRI* Magnetic resonance imaging, *AVM* Arteriovenous malformation, *DNT* Dysembryoplastic neuroepithelial tumour, *MTS/HS* Mesial temporal sclerosis/hippocampal sclerosis, *SWS* Sturge–Weber syndrome, *TSC* Tuberous sclerosis complex.

**Figure 1:**
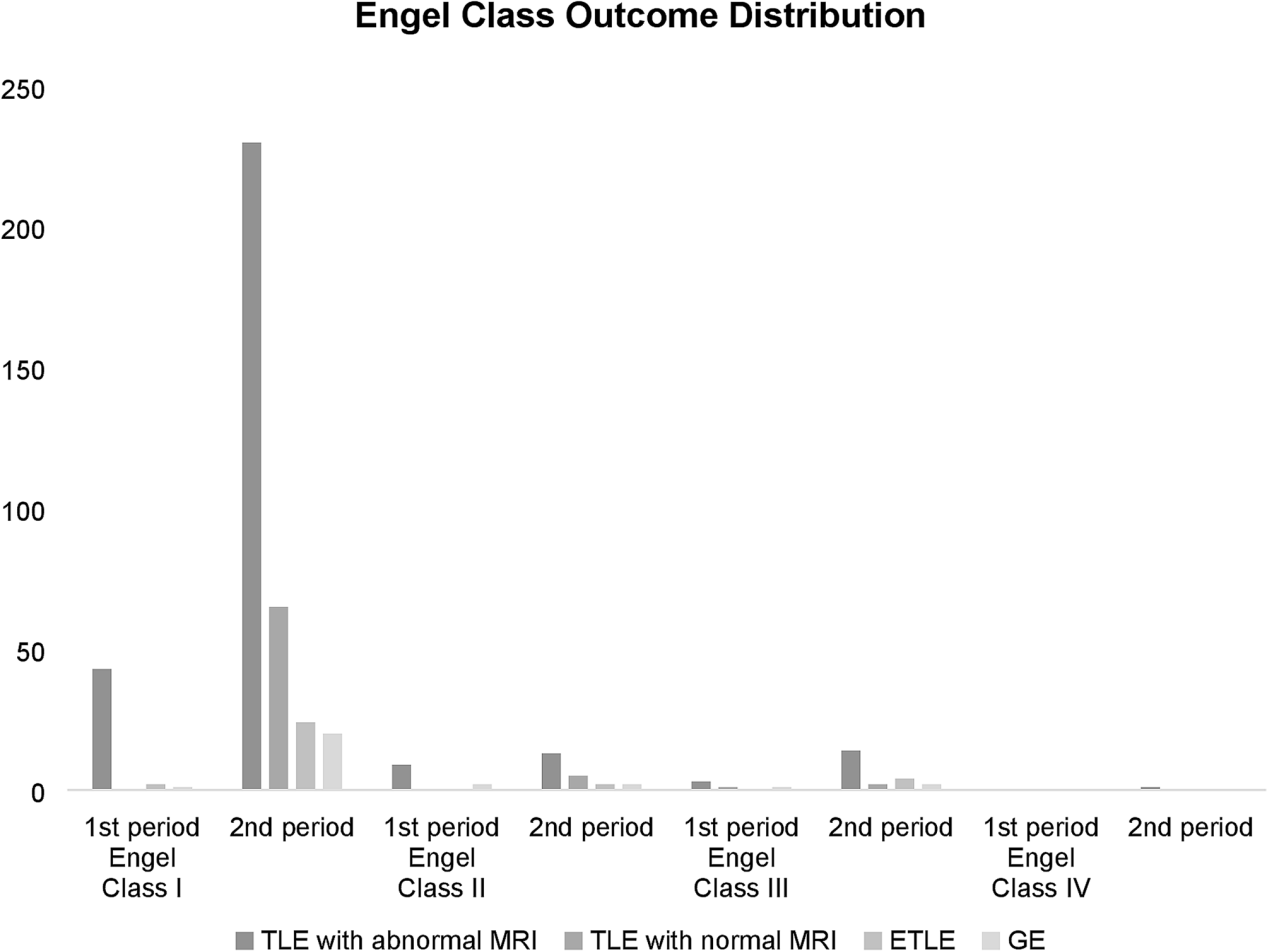
Engel class outcome distribution between the 1st and 2nd periods. Most of our patients achieved Engel Class I until the end of follow-up (December 2019).

Within the first period, 78.2%, 16.4%, and 5.5% of patients with TLE and abnormal MRI findings achieved Engel Classes I, II, and III, respectively. For the second period 84.1%, 5%, 5.4%, and 0.4% of patients achieved Classes I, II, III and IV, respectively. During the first period, 76.8%, 16.1%, and 7.1% of patients with TLE achieved Classes I, II, and III, respectively, while during the second period, 89.4%, 5.5%, 4.9% and 0.3% achieved Classes I, II, III and IV, respectively. The surgical outcomes of patients with TLE and abnormal or normal MRI findings as well as extratemporal lobe epilepsy and general epilepsy are summarized in Fig. 1 and Table 5.

**Table 5:** Engel class outcome (comparison between the 1st and 2nd periods).

	Engel Class I		Engel Class II		Engel Class III		Engel Class IV	
	1	2	1	2	1	2	1	2
TLE with abnormal MRI	43 (78.2%)	230 (84.1%)	9 (16.4%)	13 (5.0%)	3 (5.5%)	14 (5.4%)	0	1 (0.4%)
TLE with normal MRI	0	65 (90.3%)	0	5 (6.9%)	1 (100%)	2 (2.8%)	0	0
Overall Temporal	43 (76.8%)	295 (89.4%)	9 (16.1%)	18 (5.5%)	4 (7.1%)	16 (4.9%)	0	1 (0.3%)
ETLE	2 (100%)	24 (80%)	0	2 (6.7%)	0	4 (13.3%)	0	0
GE	1 (25%)	20 (83.3%)	2 (50%)	2 (8.3%)	1 (25%)	2 (8.3%)	0	0

*1* 1st period, *2* 2nd period, *TLE* Temporal lobe epilepsy, *MRI* Magnetic resonance imaging, *ETLE* Extratemporal lobe epilepsy, *GE* Generalized epilepsy.

**Figure 2:**
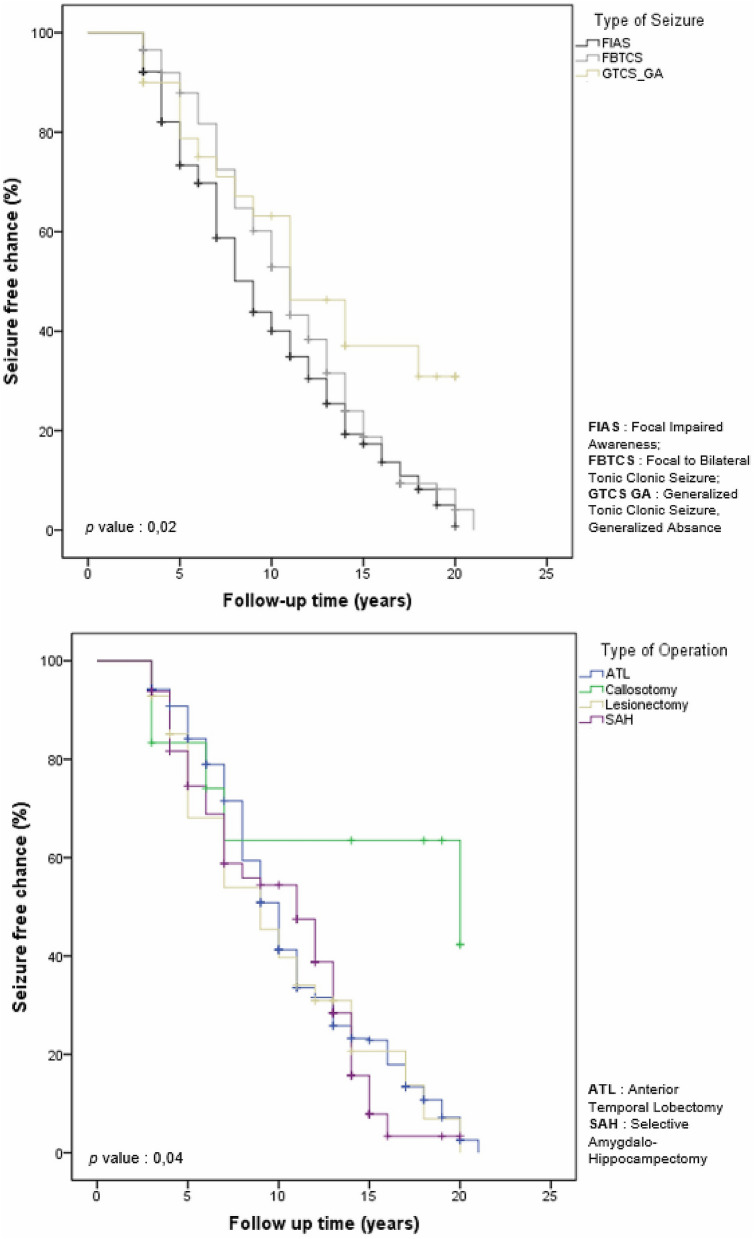
Kaplan–Meier plot predicting the chance of being seizure free at the end of follow-up. There were significant differences in the type of seizure and type of operation (*p* = 0.02; 0.04).

**Figure 3:**
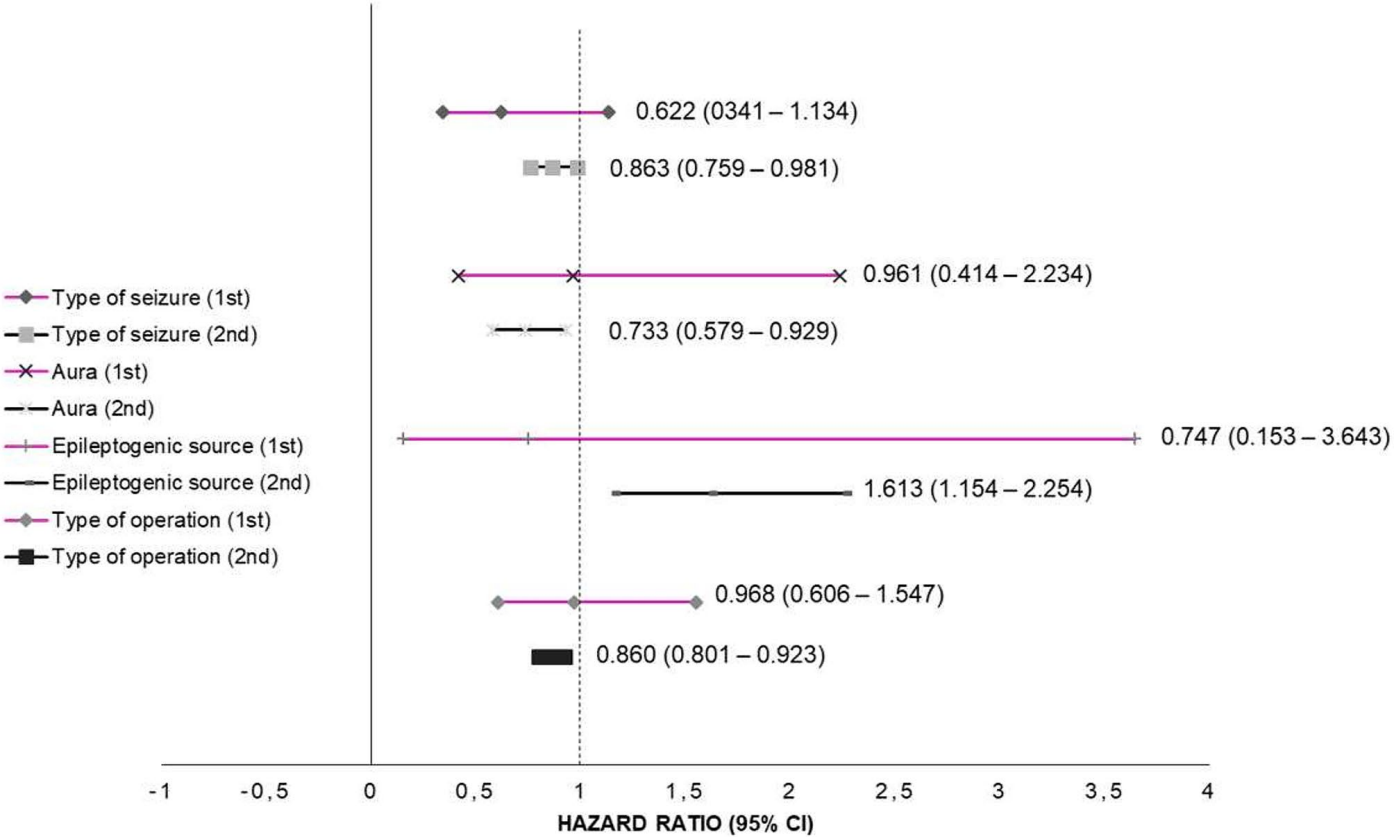
Comparison between the hazard ratio (95% CI) of each variable (1st period vs. 2nd period).

Using the chi-square test, significant differences were observed between the seizure-free and non-seizure-free groups based on seizure type, aura, epileptogenic source and type of surgery (Table 6). Based on seizure type, aura, epileptogenic source and type of surgery, the outcomes in terms of seizures were not significantly different between the first and second periods (Table 6). Kaplan–Meier analysis confirmed significant differences in the overall survival time amongst patients with FIAS, FBTCS and GTCS/GA, with respective medians of 9 (95% CI: 8.170–9.830), 11 (95% CI: 10.170–11.830), and 11 (95% CI: 7.265–14.735) years (p = 0.04), with GTCS/GA patients exhibiting the greatest survival outcomes at the end of follow-up. The overall survival time also varied significantly based on surgery, with a median of 10 years for patients who underwent ATL (95% CI: 9.364–10.636), 11 years for patients who underwent SAH (95% CI: 9.413–12.587), 20 years for patients who underwent callosotomy (95% CI: 0.000–43.958), and 9 years for patients who underwent lesionectomy (95% CI: 6.129–11.871) (*p* = 0.04), as shown in Table 6 and Fig. 2. In addition, outcomes were better for focal seizures than for general seizures, with an HR of 0.801 (95% CI: 0.705–0.909; p < 0.05), as shown in Table 6. A comparison of HRs for each variable between the 1st and third periods is presented in Fig. 3.

**Table 6:** Postoperative seizure-free outcome analysis according to the type of surgery, aura, epileptogenic source and type of operation.

Variable	*p*^a^	HR (95% CI)^b^			Median survival time in years (95% CI)	*p*^c^	3-Year FU			5-Year FU			10-Year FU		
		1st	2nd	Overall											
							N1	N2	% chance	N1	N2	% chance	N1	N2	% chance
Type of seizure	0.03*	0.622 (0.341–1.134)	0.863 (0.759–0.981)	0.801 (0.705–0.909) *		0.02*									
FIAS					9 (8.170–9.830)		22	258	92.1	70	185	73.4	151	95	40.0
FBTCS					11 (10.170–11.830)		7	195	96.5	24	172	87.9	92	102	52.9
GTCS/GA					11(7.265–14.735)		3	27	90.0	6	21	78.8	14	11	46.3
Aura	0.006*	0.961 (0.414–2.234)	0.733 (0.579–0.929)	0.799 (0.637–1.002)		0.238									
Aura persists					10 (9.215–10.785)		27	390	93.5	83	306	79.1	213	169	45.1
Aura negative					11 (9.658–12.342)		10	162	94.2	33	128	79.9	77	74	50.6
Epileptogenic source	0.006*	0.747 (0.153–3.643)	1.613 (1.154–2.254)	1.210 (0.871–1.681)		0.882									
Temporal					10 (9.254–10.746)		29	483	94.3	97	385	80.2	253	216	47.0
Extra temporal					9 (6.163–10.699)		8	69	89.6	19	49	73.3	37	27	44.6
Type of operation	0.003*	0.968 (0.606–1.547)	0.860 (0.801–0.923)	1.046 (0.978–1.118)		0.04*									
ATL					10 (9.364–10.636)		18	292	94.2	47	243	84.2	167	112	41.3
SAH					11 (9.413–12.587)		14	211	93.8	55	158	74.6	111	96	47.5
Callosotomy					20 (0.000–43.958)		2	10	83.3	3	8	74.1	5	2	42.3
Lesionectomy					9 (6.129–11.871)		3	39	92.9	12	24	68.1	22	14	39.7

*FU* Follow-up, *FIAS* Focal impaired awareness seizure, *FBTCS* Focal to bilateral tonic clonic seizure, *GTCS/GA* Generalized tonic clonic seizure generalized absence, *ATL* Anterior temporal lobectomy, *SAH* Selective amygdalohippocampectomy, *1st* 1st period, *2nd* 2nd period, *HR* Hazard ratio, *CI* Confidence interval, *N1* Number at risk, *N2* Number of remaining cases, *% chance* % chance of being seizure free at the end of follow-up.

^a^Chi-square test.

^b^Cox regression analysis of seizure free.

^c^Kaplan–Meier analysis with the log-rank test.

*Statistically significant at *p* < 0.05.

Postoperative outcomes were analysed between the 1st and 2nd periods. At 5 years after surgery, 47/65 patients (72.3%) in the 1st period and 424/524 patients (80.9%) in the 2nd period were seizure free. Eighteen of 65 patients in the 1st period (27.7%) and 100 of 524 patients (19.1%) still had seizures at the 5-year follow-up. There was no significant difference in the 5-year follow-up outcome between the 1st period and 2nd period (p = 0.102) (Table 7).

**Table 7:** Postoperative outcome analysis between the 1st and 2nd periods.

	5-year follow-up		*p*
	Seizure free	Seizure	
1st period	47 (72.3%)	18 (27.7%)	0,102
2nd period	424 (80.9%)	100 (19.1%)	

## Discussion

This report presented long-term outcomes of a large cohort of epilepsy patients who underwent surgery (589 people) with at least 2 years of follow-up, covering nearly three seizure-free quarters. In addition, this report strengthened our previous study epilepsy surgeries conducted in Indonesia, the most populous country in Southeast Asia^8^. Previous authors have described the establishment of this surgical service as feasible and cost-effective in other developing countries, including India, Lebanon, and Colombia, with comparable outcomes to those in developed countries^9–14^. However, during its initial development in 1999, epilepsy surgery was unknown to most persons in Indonesia; hence, the staff worked hard to establish support from the medical community, especially the neurological society.

Therefore, the first period (1999–2004) featured only selected patients who required simple diagnostic procedures, ensuring the achievement of an excellent outcome and near-zero morbidity. Scalp EEG, MRI and seizure semiology were the primary evaluation tools for surgery; hence, the criteria used reserved the surgery for remediable syndromes, characterized by well-understood pathophysiologies. Additionally, the natural history was medically refractory or progressive, following failure of a major first-line AEDs.

During this period, all preoperative evaluations were conducted non-invasively, and surgery was offered as an excellent opportunity to accomplish the complete elimination of seizures. Furthermore, the eligible candidates for excisional procedures and surgery usually possessed localized and generalized epilepsy syndromes, respectively, as mesial TLE features neocortical epilepsy caused by discrete, easily resectable lesions.

### Surgical outcomes

Engel Class I was achieved in 78.2% and 84.1% of patients with TLE and abnormal MRI findings during the first and second periods, respectively. This result was similar to those of previous reports from studies conducted in both adults and children, although freedom from seizures was more common after resection with MTS (60–90%)^15–17^. Meanwhile, ATL conducted by others within the past 15 years who attained freedom from seizures on approximately 70–80% of adults and children with TLE^18,19^, and resected TLE surgery has been validated by class I evidence^20^. Among two randomized controlled trials, the first reported that 58% of drug-resistant TLE patients who had undergone ATL were seizure free at 1 year compared to the 8% who had continued best drug therapy^20^. After 5 years of postoperative follow-up, 47/65 patients in the 1st period (72,3%) and 424/524 patients in the 2nd period (80,9%) achieved a seizure-free status, with no statistically significant difference between the 1st and 2nd period outcomes (Table 7 and Fig. 3). Furthermore, the limited resources available were used to achieve similar results.

Engel’s classification was adopted to analyse outcomes. One study^21^ reported a 60% response rate on average 7 years after temporal surgery, which showed the absence of seizures in 48% of patients from the date of surgery. This outcome covers 40% of all seizure-free individuals in this cohort 10 years following anterior temporal resection based on the survival analysis estimation (Table 6). Another postsurgery study reported that 43% of patients were seizure free after FIAS after a mean 7-year follow-up, while in other studies^22,23^, between 41 and 63% of patients with or without FIAS were seizure free after being monitored for 5–10 years after temporal surgery.

During this cohort study, the probability of being seizure free (no seizures with loss of awareness) was 92.1% within a 3-year period, which decreased to 40% after a decade. A study on consecutive people who underwent temporal resection for hippocampal sclerosis estimated seizure-free success rates at the 2- and 5-year intervals that were lower than those in the current study^24^ (94.3% at 3 years and 80% at 5 years) (Table 6). Unsurprisingly, others^25^ associated the short interval between surgery and recurrence of the first seizure with a longer term prognosis than the interval.

It has been established that patients who undergo extratemporal resection have a greater probability of recurrence than those who undergo anterior temporal resection. In addition, similar findings were reported in a long-term postsurgical follow-up study^14^, where individuals who underwent temporal resection had an increased chance of being seizure free.

This favourable outcome has been reported in previous studies^26,27^, as the overall seizure-free rate after anterior temporal resection was 76.8% within the first period and 89.4% in the second period for patients with temporal lobe epilepsy or hippocampal sclerosis.

A postoperative outcome study^28^ on patients with pathological changes identified on preoperative MRI showed 2-year remission in 80% of respondents with tumours and vascular malformations, 58% of those with hippocampal sclerosis, and 29% with normal MRI findings. However, the assessment^29^ of people who underwent anterior temporal resection showed the inability for changes in the underlying pathology to determine the time of subsequent seizures. Additionally, the presence of a ganglioglioma or dysembryoplastic neuroepithelial tumour and the absence of dysplasia have been associated with good postoperative control^30^. Moreover, the majority of patients treated in the current study included those with hippocampal sclerosis (56.16%), while 17.37% had normal MRI findings, and 84.9% were free of seizures, as shown in Table 4.

The probability for patients to maintain this positive outcome increased with an increasing number of seizure-free years (Table 6). This finding is supported by the results of a study on the outcome after temporal surgery, where individuals who received successful treatment for any 1 year or those with only SPS had a 90% probability of having no seizures in the subsequent year^23^. However, those who achieved 2 successive years without seizures had a greater chance of having a seizure the subsequent year (94%).

Intraoperative monitoring in epilepsy surgery involves two aspects: monitoring cortical activity and identifying the epileptogenic zone (focus) minimal amount of tissues to be resected in a bid to control seizures. Additionally, there is a need to screen for a functionally important cortex, which should be spared during the resection process. Furthermore, the use of an intraoperative evaluation in the verification of epileptogenic zones is often based on electrocorticogram (ECoG) recordings, which were conducted in 45 patients. Additionally, the Wada test modified with propofol was performed in one patient. This was not routinely administered because of the difficulty associated with the test and the availability of amobarbital in Indonesia.

### Strategies to overcome problems in centres with limited resources

This study showed attempts to address several problems during the establishment of an epilepsy surgical centre. One of most challenging was the poor availability and feasibility of the service, which consequently created an enormous "surgical treatment gap" between the number of potential beneficiaries and the actual surgery performed, which was wide in a low-and middle income society^31–34^. Additionally, execution is difficult on an isolated basis, as the procedure should be performed within an uninterrupted, well-defined program that uses a systematic approach with established criteria for patient selection, surgery, and follow-up^7^. This decision-making step requires a multidisciplinary approach in which different investigators cooperate to understand epileptology^35^. Therefore, a successful outcome depends greatly on the teams’ willingness to identify suitable candidates with an epileptogenic zone that is clearly established using locally available equipment and knowledge without risking patient safety^36,37^. This also involves acquiring knowledge on when not to perform the operation and requesting additional evaluation, which is important in the identification of beneficiaries with the available resources^38^.

Several core strategies have been implemented to improve the services rendered. (1) Recruiting a trained epileptologist who is knowledgeable in all aspects of seizure control. Furthermore, the ability to organize and manage a team is probably the most important element in establishing a successful epilepsy care centre. This is achievable by seeking assistance from well-established service providers in developed countries due to the poor feasibility of self-training through trial and error within a limited setting. However, the epileptologist in our facility (Dr. Aris Catur Bintoro) received training in the well-respected National Epilepsy Center, Shizuoka Institute of Epilepsy and Neurological Disorder, Japan. (2) Incorporating expert neurosurgeons, both individually and as a group. The team in this study comprised two neurosurgeon, both trained in Japan. However, active enrolment in an epilepsy fellowship program within advanced centres and obtaining appropriate training are assumed to be effective for long-term disease management. (3) Seeking EEG technicians to undergo relevant training; hence, the epileptologist always has three qualified nurses. In places without access to EEG technician training programs, registered nurses with additional medical knowledge and motivation to participate are optimal training recipients (under the epileptologist’s supervision). (4) Granting access to basic investigation modalities (i.e., MRI and EEG or, preferably, video-EEG) through public health support systems. In addition, MRI for the evaluation of patients with refractory epilepsy should be performed using a special temporal lobe protocol that is then interpreted by an experienced radiologist accustomed to identifying hippocampal sclerosis. Collaborations were established with the radiology department to create an epilepsy protocol that is appropriate for epilepsy imaging, which led to the assignment of one expert to specifically work with the epilepsy surgery team. The centre was also equipped with a long-term video-EEG monitoring device in 2011, alongside reasonable customer and technical support, followed by the separate use of a video tape recorder and time synchronization with the EEG machine. (5) Developing realistic preoperative protocols featuring good history collection is key to selecting appropriate surgical candidates. This challenge was solved by developing and routinely using a protocol that was designed to minimize the risk of missing important clinical details, including seizure type and frequency, associated disabilities, and the impacts of uncontrolled seizures. The next step after collecting the relevant information was to provide the patient details to the epilepsy surgery committee, which comprised the epileptologist, two neurosurgeons and the neuroradiologist. This multidisciplinary approach towards the management of individuals with medically refractory epilepsy was unique to the centre under investigation.

Since 2010, these programs have provided excellent care for patients with epilepsy, while more centres are currently in the developmental phase. This has been significantly hampered by limitations in available resources and difficulties faced in attempts to access epileptologists, staff, and equipment. Following extensive discussions amongst all team members, a decision was reached regarding three possible surgical scenarios in patients with medically refractory epilepsy. These include: a. Lesional epilepsy resections: performed in instances where a brain lesion is identifiable by MRI, despite discordant semiology and interictal EEG evaluations with the lesion, and when the surgery procedure has no significant risks; b. Anterior temporal lobectomy in temporal lobe epilepsy: conducted when there is a display of unilateral mesial temporal sclerosis and semiology as well as when interictal EEG is concordant with the MRI findings; and c. Palliative surgery (corpus callosotomy): carried out when patients demonstrate intractable generalized seizures and are not considered candidates for focal resection. This is also contemplated for those with refractory frontal lobe seizures and a tendency to be adequately localized^39^.

## Conclusions

This report sought to share the experiences of organizing the epilepsy surgery program in Indonesia. Our experience suggests that it is possible to initiate the surgical program even under imperfect circumstances, such as limited modalities for presurgical evaluations. We emphasized the utilization of stringent and selective criteria to reserve surgeries for remediable syndromes characterized by well-understood pathophysiologies, such as drug-resistant TLE with typical symptoms and the concordance of interictal EEG and MRI findings. These patients were identified as good candidates for the commencement of epilepsy surgery in countries with limited resources based on positive results obtained from our centre.
